# Assessment of sperm chromosomal abnormalities using fluorescence in situ hybridization (FISH): implications for reproductive potential

**DOI:** 10.1007/s10815-024-03224-4

**Published:** 2024-08-20

**Authors:** Francesca Paola Luongo, Eugenia Annunzi, Francesca Girolamo, Giuseppe Belmonte, Rosetta Ponchia, Paola Piomboni, Alice Luddi

**Affiliations:** Department of Molecular Medicine and Development, University of Siena, 53100 Siena, Italy

**Keywords:** Sperm, FISH, Aneuploidies, Semen parameters

## Abstract

**Purpose:**

Chromosomal abnormalities play an important role in male infertility, which is becoming a significant issue in human fertility. Aim of this study was to evaluate the incidence of spermatic aneuploidies and diploidies in human sperm, according to semen parameters.

**Methods:**

We performed semen analysis according to the 6th edition of WHO criteria in 50 male subjects; samples were divided into normozoospermic (n = 23) or those with altered seminal parameters (n = 27). To assess chromosomal numerical alterations of sperm, fluorescence in situ hybridization (FISH) was used.

**Result:**

A significant increase in aneuploidies and diploidies was observed in samples with altered seminal parameters. Furthermore, stratifying this group, we observed a significant increase in aneuploidies and total abnormalities in oligozoospermic, asthenoteratozoospermic (AT), and oligoteratoasthenozoospermic (OAT) samples compared to normozoospermic.

**Conclusion:**

Our results showed the correlation between altered seminal parameters and numerical chromosomal abnormalities, confirming that sperm FISH analysis could be an additional clinical tool to assess reproductive potential in infertile males. Moreover, our results point to the importance of updating the normality ranges for detecting chromosomal aneuploidies using FISH.

**Supplementary information:**

The online version contains supplementary material available at 10.1007/s10815-024-03224-4.

## Introduction

Sperm chromosomal abnormalities play a significant role in male infertility, accounting for 2–14% of cases of male infertility. Indeed, both numerical and structural chromosomal aberrations in sperm are major contributors to pregnancy loss [1], perinatal death, congenital malformations, mental retardation, and behavioural anomalies [2]. Among the numerical abnormalities, errors during mitosis or the first or second meiotic divisions of germ cells may lead to the formation of aneuploid gametes, in which extra or missing chromosomes (either the autosomes and/or the sex chromosomes) are present [3]. During meiotic phases of spermatogenesis, two mechanisms can cause chromosome segregation errors: the primary process responsible is non-disjunction, which leads to the formation of gametes with either an absence or an excess of chromosomes (nullisomic and disomic gametes). Furthermore, anaphase lag can yield sperm containing solely nullisomic chromosomes. The incidence of chromosomal aberrations was reported to be increased in sperm from men with abnormal semen parameters [4, 5]. Gametes of infertile men show a higher rate of chromosome abnormalities than the general population [6]; on the other end, sperm with normal morphology does not correlate with normal haploid chromosomal assets [7].

Infertility represents a significant health concern, affecting up to 17.5% of couples of reproductive ages [8], and approximately 50% of these cases a male factor is identified. To address this, an enhanced fluorescence in situ hybridization (FISH) protocol has been developed for analyzing the chromosomal composition of sperm in infertile men [9]. This protocol is now widely recognized as the standard approach for detecting chromosomal abnormalities in this context. In recent decades, progress in FISH techniques, employing chromosome-specific DNA probes, has streamlined the rapid screening of germ cells for chromosomal aberrations. Consequently, a large number of studies including FISH technique in spermatozoa have been published and it has been established that a significant percentage of individuals with infertility have increased chromosomal abnormalities in sperm [10, 11]. In the latest WHO edition [8], the editors acknowledge the growing awareness of genetics-related male infertility, particularly the various forms of sperm chromosomal abnormalities and gene mutations, addressing the utility of FISH testing as a cytogenetic diagnostic tool in the evaluation of chromosomal aberrations, reporting the incidence of chromosomal sperm disomy in fertile men. However, the authors do not adequately address the indications for sperm genetic testing, and it is unclear how the tests can be used to guide the management of infertile couples in clinical practice. In fact, currently this analysis is applied to patients with altered sperm parameters and couples with a clinical history of recurrent abortion or repeated implantation failure [12].

Noteworthy, lifestyle habits and environmental exposure to pollutants induced a significant decline in sperm number by almost half in the last few decades [13, 14]. While the utility of FISH in detecting chromosomal aneuploidies is well-established and widely accepted, we believe it is crucial to address the shifting baseline of what is considered "normal" in light of changing semen parameters. Therefore, the aim of this analysis was to highlight the correlation between chromosomal abnormalities in spermatozoa and the quality and characteristics of sperm. This examination comes at a time when male factors are increasingly being identified in fertility cases, with numbers expected to grow due to a global decline in human sperm quality.

## Materials and methods

### Participants

This study was conducted on samples from a cohort of 50 males (age: 32–50 years; mean: 41.2 ± 4.9 years) undergoing semen analysis for fertility evaluation at the Unit of Medically Assisted Reproduction, Siena University Hospital, from 2021 to 2023. The study protocol received approval from the Ethical Committee of the Siena University Hospital (approval ID: CEAVSE, protocol number 18370, 2/10/2020); before participating, all subjects gave their written informed consent. A comprehensive clinical history was obtained for all participants, and subjects with possible preexisting causes of male infertility, such as varicocele, cryptorchidism, or endocrine disorders, were excluded. All individuals had a normal karyotype, and no history of chemotherapy, radiotherapy, or chronic illness. Among the fifty subjects, twenty-one derive from a situation of couples of polyabortion, eighteen from missed and failed PMA techniques, seven from biochemical pregnancies and the remaining four in search of a pregnancy.

### Sample collection and semen analysis

Semen samples were obtained from all patients by masturbation, after 3–5 days of sexual abstinence. After complete liquefaction of the sample at room temperature, the macroscopic and chemical-physical analysis was performed [15]. A spermiogram was carried out according to the last edition of the World Health Organization criteria [8]. Samples were categorized into normozoospermic and altered seminal parameters groups based on semen characteristics, with the latter further classified into various seminal phenotypes, including oligozoospermic, oligoteratoasthenozoospermic (OAT) and other phenotypes not including a reduction in sperm count and morphology (asthenoteratozoospermic, AT).

### Fluorescent in situ hybridization (FISH)

The cytogenetic analysis FISH was performed to evaluate the chromosomal numerical alterations of sperm following a published protocol with some modification [16]. Briefly, the semen sample was resuspended with hypotonic solution KCl 0.075 M an incubated at 37 ºC, fixed in Carnoy solution (3:1 methanol-acetic acid), and smeared on glass slides. Afterward, sperm were dehydrated and nuclei decondensed in a dithiothreitol (DTT) solution, the times vary among semen samples features. Subsequentially, slides were denatured in 70% formamide /2X saline sodium citrate buffer (SSC, Sigma-Aldrich, Saint Louis, MO, USA) at 73 °C 5 min and hybridized at 37 °C in a humid chamber overnight. The chromosomes were labelled using Chromosome enumeration probes (CEP, Vysis, IL, USA) α-satellite DNA probes for chromosomes X, Y and 18, directly labeled with different fluorochromes (Supplementary Table 1). The probe mix was denatured for 5 min at 75 °C in a water bath. Post-hybridization samples were washed to remove any unbound DNA probe. Finally, slides were counterstained with 4,6-diamidino-2-phenylindole (DAPI-II; Vysis, Abbott Molecular Inc, IL, USA). Fluorescence images were acquired by the Leica AF6500 Integrated System for Imaging and Analysis (Leica Microsystems, Wetzlar, Germany), equipped with the LAS AF software. An average of 5000 sperm nuclei per sample were analyzed.

### Aneuploidy and diploidy assessment

**Fig. 1:**
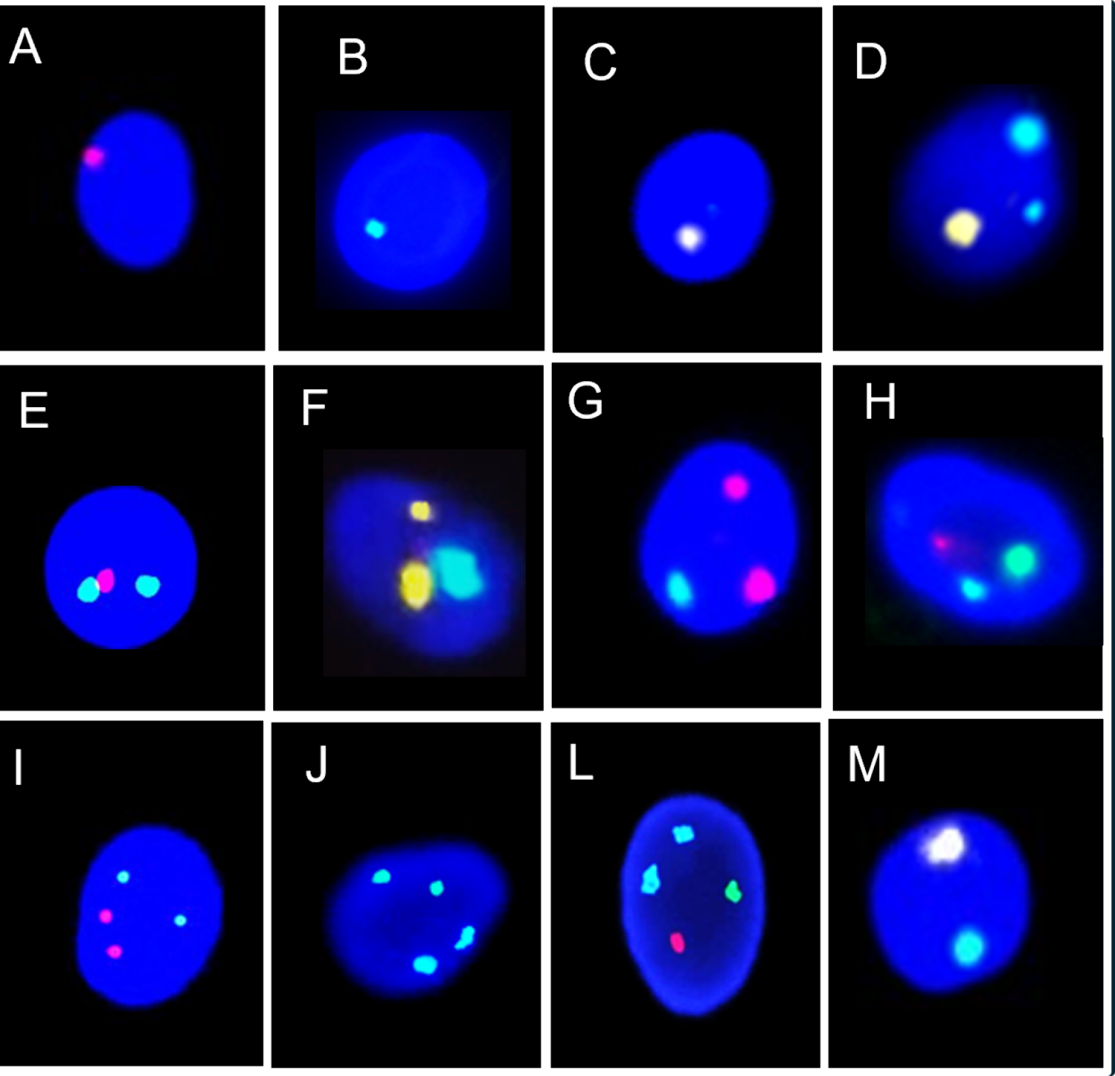
Representative images of different FISH signal patterns used to assess sperm chromosomal abnormalities. Nullisomies, where one chromosome signal is absent (**A**-**C**). Disomies, featuring two signals from the same chromosome and one from another (**D**-**H**), demonstrate distinct signal separation. Diploidies, indicating two signals from each chromosome (**I**-**L**), reflect a consistent pattern across the set. Normal chromosomal asset (M). Y (green), X (red), 18 (yellow), Magnification: 6300 x

The FISH signals assessment and data analysis were conducted following strict criteria, assessed by two independent operators. Only sperm heads showing a regular outline and well-defined limits were evaluated. Disomies and diploidies were confirmed when all signals were of the same intensity, dimension, and shape. Nullisomies (X0; Y0; 180; Fig. 1, A-C) were defined when no signal was detected for only one chromosome in a set, in the presence of other chromosome signals. Disomies (1818Y;1818X;18YY;18XX; 18XY; Fig. 1, D-H) were defined as two separate signals for the same chromosome and one signal for the other chromosome in a set. Diploidies (1818YY;1818XX; 1818XY; Fig. 1 I-L) were defined as two signals from each chromosome in a set.

### Statistical analysis

Data were analyzed using one-way analysis of variance or non-parametric testing (when the data was not normally distributed, and the error variance was unequal). A Mann–Whitney test was used for a comparison between controlled and altered precisely normozoospermic and subjects with altered seminal parameters. Instead, a Kruskal–Wallis test was used, for a multiple comparison between control and various seminal phenotypes for the calculation of statistical significance. All tests were conducted using GraphPad Prism v.8.0.2. (Boston, MA, USA), and results with *p* < 0.05 were considered significant.

## Results

### Sample characteristics according to last WHO edition

The cohort was divided into two groups; (i) samples with normal seminal parameters (normozoospermic n = 23) and (ii) samples with altered seminal parameter (n = 27). The demographic data and seminal parameters have shown a significant reduction in the number of sperm (*p* < *0.0001*), motility (*p* = *0.004*), and morphology percentage (*p* =  < *0.0001*) in samples with altered seminal parameters compared to the normozoospermic group. However, we have not observed significant differences in age and ejaculate volume (see Table 1).

**Table 1:** Comparison between normozoospermic and samples with altered seminal parameters. The data are expressed with mean ± SD

Seminal parameters	Normozoospermic (n = 23)	Altered seminal parameters (n = 27)	*p* value
Age	39.4 ± 5.0	42.0 ± 5.9	0.099
Volume (mL)	3.4 ± 1.4	2.8 ± 1.6	0.151
Number of spz (× 10^6^) /mL	64.2 ± 30.5	45.9 ± 57.5	0.005***
Total number of spz (× 10^6^)	204 ± 96.5	100 ± 142	< 0.0001****
Motility (%)	60.3 ± 10.4	49.8 ± 15.0	0.004***
Morphology (%)	7.0 ± 2.6	2.8 ± 2.5	< 0.0001****

### Incidence of aneuploidy and diploidy in normozoospermic vs altered seminal parameter

FISH cytogenetic analysis was performed for all samples, which allowed identification of all numerical alterations in chromosomes 18, X, and Y. As shown in Table 2, we observed a significant increase in the total frequency of chromosomal alterations (sum of diploidies and aneuploidies) in samples with altered seminal parameters (0.82 ± 0.24) compared with normozoospermic (0.66 ± 0.24; *p* = *0.028*). When we look at the diploidies, we highlighted a significant increase of the diploidies percentage in samples with altered seminal parameter compared to those classified as normozoospermic (*p* = *0.037*). Furthermore, by summing the frequencies of nullisomies and disomies, our findings revealed a heightened total number of aneuploidies in samples exhibiting altered seminal parameters (0.56 ± 0.16) compared to normozoospermic samples (0.46 ± 0.14; *p* = *0.025*). Additionally, our results indicated no significant difference in the mean percentage of nullisomies, while a significant increase in sperm disomies was observed in individuals with altered seminal parameters (*p* = *0.036*).

**Table 2:** Incidence of aneuploidies, diploidies and total of chromosomal anomalies in normozoospermic *vs.* altered seminal parameters. The data are expressed with mean ± SD

	Aneuploidies (%)			Diploidies (%)	Total of chromosomal anomalies (%)
	Nullisomies	Disomies	Total aneuploidies		
Normozoospermic	0.17 ± 0.11	0.27 ± 0.06	0.46 ± 0.14	0.20 ± 0.10	0.66 ± 0.24
Altered seminal parameters	0.22 ± 0.12	0.33 ± 1.0	0.56 ± 0.16	0.26 ± 0.13	0.82 ± 0.24
*p* value	0.14	0.036*	0.025*	0.037*	0.028*

Based on these findings, we conducted a more in-depth analysis of disomy frequency for each chromosome included in the study. As outlined in Table 3, a significant increase in the 18/18 disomy was identified within the group exhibiting altered seminal parameters compared to the control group (*p* = 0.016).

**Table 3:** Incidence of chromosome 18, X and Y in normozoospermic and samples with altered seminal parameters. The data are expressed with mean ± SD

	Disomies			
	18/18	X/X	Y/Y	X/Y
Normozoospermic	0.08 ± 0.03	0.09 ± 0.03	0.06 ± 0.04	0.05 ± 0.03
Altered seminal parameters	0.10 ± 0.04	0.09 ± 0.04	0.06 ± 0.03	0.07 ± 0.05
*p* value	0.016*	0.925	0.488	0.231

Although there was a visible trend towards higher values in X/Y disomy was observed in samples from individuals with altered parameters, statistical significance was not reached for the other analyzed disomies.

### Incidence of aneuploidies and diploidies based on individual seminal parameters

**Fig. 2:**
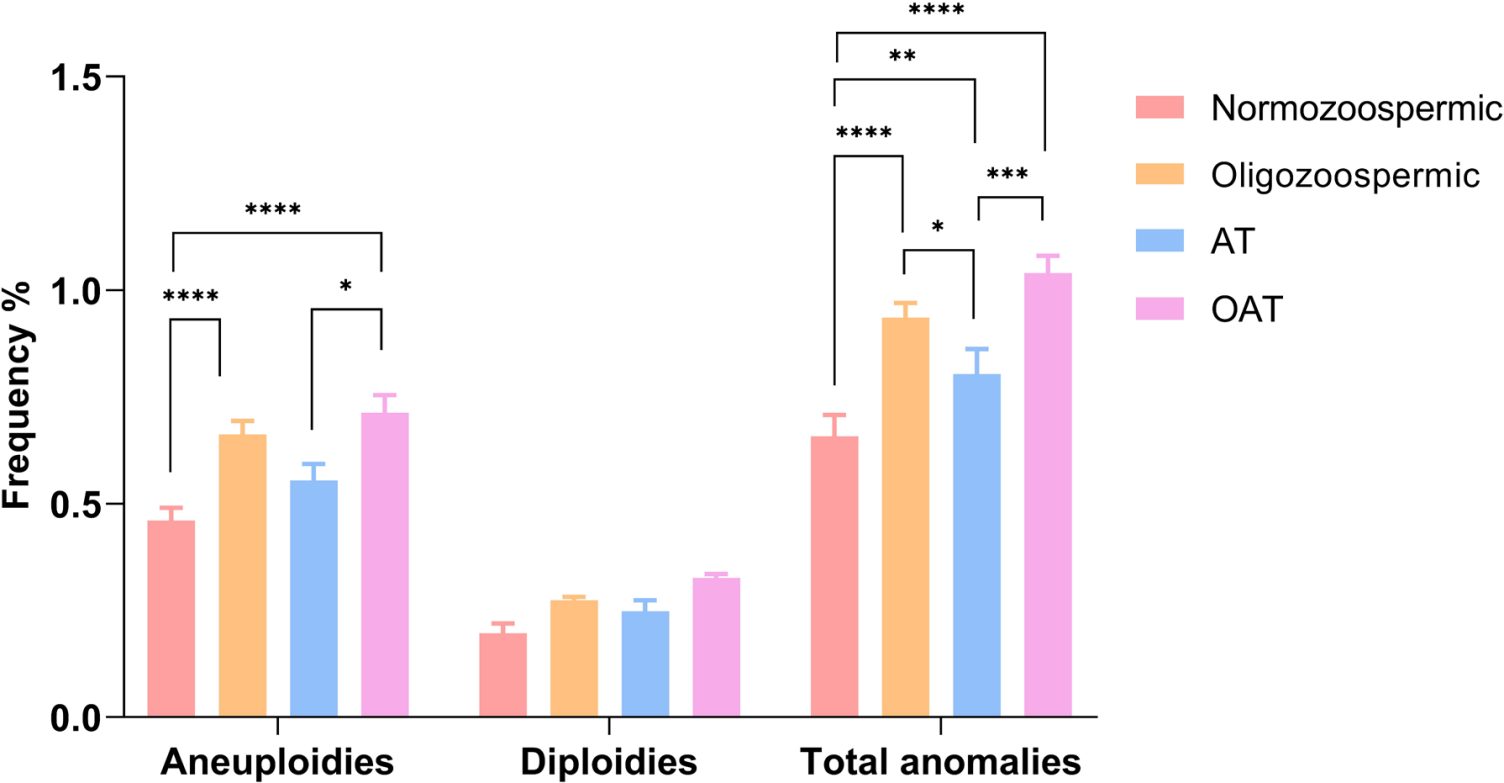
Mean frequencies of aneuploidies, diploidies and total of chromosomal anomalies in normozoospermic and samples classified in oligozoospermic, AT and OAT. Significant differences are indicated (Bonferroni correction *p < 0.05; **p < 0.01; ***p < 0.001; ****p < 0.0001)

To stratify our data, the subjects with altered seminal parameters were divided in oligozoospermic, AT and OAT. When considering the total of chromosomal anomalies, we noted a significant increase of frequency in oligozoospermic (0.936), AT (0.803) and OAT (1.040) samples compared to normozoospermic ones (0.657) (Fig. 2 and Supplementary Table 2). This increase was particularly pronounced in samples exhibiting a decrease in sperm count compared to the occurrence of other seminal phenotypes.

When analyzing aneuploidies, our findings revealed a significant increase in the total frequency of aneuploidies in oligozoospermic and OAT samples compared to the control group. Also in this case, we noted a significant increase in the OAT group compared to AT samples. However, regarding diploidies, no significant differences were observed in our study cohort.

**Fig. 3:**
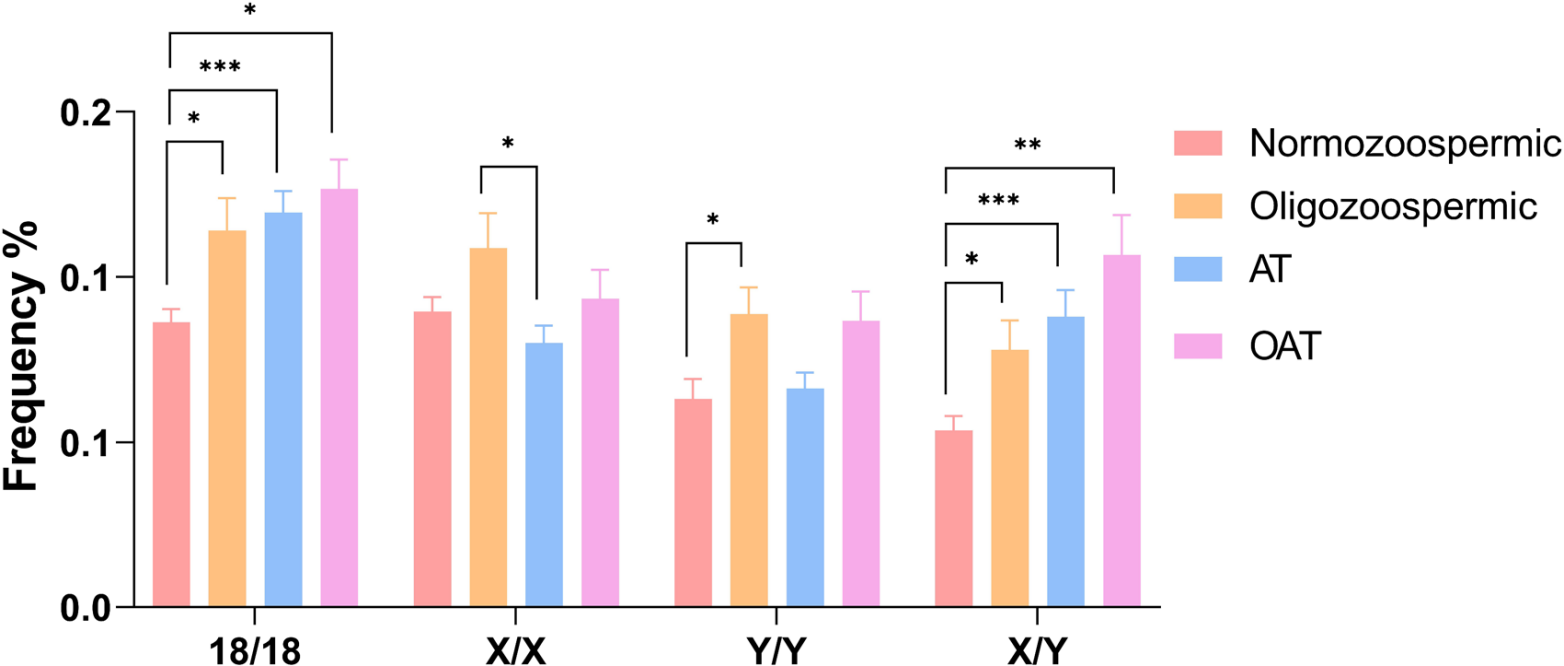
Mean frequencies of disomies in normozoospermic and samples classified in oligozoospermic, AT and OAT. Significant differences are indicated (Bonferroni correction *p < 0.05; **p < 0.01; ****p < 0.0001; ***p < 0.001)

Upon analyzing the frequency of disomies for each chromosome, we observed a significant increase of 18/18 and X/Y frequency in all categories of altered semen parameters compared to normozoospermic samples (Fig. 3 and Supplementary Table 3 for details). Furthermore, for chromosome Y disomy, a significant increase was observed in the oligozoospermic group compared to normozoospermic samples.

## Discussion

The present study provides an in-depth analysis on the association between seminal parameters and sperm chromosomal abnormalities. Our cohort analysis revealed a significant increase in the total frequency of chromosomal alterations, characterized by both diploidies and aneuploidies, compared to normozoospermic samples. This finding is in agreement with the scientific literature, demonstrating a correlation between seminal parameters and chromosomal aneuploidies. In particular, Tempest (2011) [17] discussed how meiotic recombination errors, which can lead to chromosomal aneuploidies in sperm, may be influenced by seminal parameters. Aberrations in sperm count, motility, and morphology could disrupt the meiotic process, increasing the likelihood of chromosomal abnormalities in sperm. Indeed, chromosomal abnormalities are found to be closely associated with morphological alterations, such as macrocephaly and the presence of supernumerary nuclei and flagella [18, 19].

The present study also provided interesting insights about the prevalence of the 18/18 disomy. We found it significantly increased in samples from subjects exhibiting altered seminal parameters compared to the control group. Similarly, Martin et al. (2003) [20] reported elevated levels of chromosome 18 disomy in subjects with asthenozoospermia and oligozoospermia compared to normozoospermic ones. Moreover, a meta-analysis by McAuliffe et al.(2012) [21] pooled data from multiple studies to assess the relationship between semen parameters and sperm chromosomal abnormalities, showing a significant positive correlation between abnormal semen parameters and increased rates of chromosome 18 disomy, further corroborating the findings observed in our study.

The stratification of subjects with altered seminal parameters into oligozoospermic, asthenoteratozoospermic (AT), and oligoasthenoteratozoospermic (OAT) groups allowed for a more nuanced analysis of chromosomal anomalies and their association with specific semen phenotypes. Our findings revealed a significant increase in the frequency of chromosomal anomalies across all three groups compared to normozoospermic samples, with the highest incidence observed in the OAT group. This result aligns with previous studies that have reported an elevated prevalence of chromosomal abnormalities in infertile men with various semen abnormalities. In particular, a study by Tempest and Griffin (2004) [22] observed a higher frequency of chromosomal anomalies, including aneuploidies and diploidies, in samples showing oligozoospermia, asthenozoospermia, and teratozoospermia compared to samples from fertile subjects. Similarly, a study by Carrell et al. (2003) [23] analyzed sperm chromosomal abnormalities in men with oligoasthenoteratozoospermia and found a significant increase in the incidence of aneuploidies and diploidies compared to men with normozoospermia. Furthermore, a meta-analysis by Zhu et al. (2022) [24] revealed a significant positive correlation between oligozoospermia, asthenoteratozoospermia and oligoasthenoteratozoospermia, and the incidence of chromosomal aneuploidies and diploidies, supporting our observation of increased chromosomal anomalies in samples with altered seminal parameters. Interestingly, our analysis showed a significant negative relationship between the reduction of sperm count and the presence of chromosomal aberrations. This aligns with previous studies have demonstrating that severely oligospermic individuals exhibit an increase of immature spermatozoa and showed a higher incidence of chromosomal aneuploidies compared to normospermic fertile men [11, 20, 25–27].

The latest edition of the World Health Organization's laboratory manual for the examination and processing of human semen [8] has also emphasized the strong association between abnormal semen parameters and an increased risk of chromosomal aneuploidies in sperm. With the revisions introduced by WHO 2021 in the threshold values for semen analysis, this study presents a significant opportunity to revisit and compare the findings of a prior investigation conducted by our group [19], a decade later. In that study, we evaluated the incidence of chromosomal segregation errors (in autosomes 13, 18, 21, and sex chromosomes X and Y) in a cohort similar to the one examined in the current research. The present study confirmed the significant increase in 18/18 and X/Y disomies in gonosomes as previously reported [19], although we did not observe the pronounced differences noted in the study published a decade ago. We cannot exclude that this discrepancy could potentially be attributed to a decline in seminal fluid quality over the years, resulting in normozoospermic samples that deviate from the previously observed "normal" characteristics. This intra-laboratory control holds significant importance, as variations in FISH technique efficiency have been reported among different laboratories. These variations stem not only from individual factors such as the quantity of spermatozoa on the slide and seminal phenotypes but also from specific technical considerations like the extent of nuclei decondensation, the types of probes used, and the efficacy of hybridization. Consequently, it is imperative to standardize these variables and maintain consistency, particularly within the same laboratory, to ensure accurate interpretation and comparison of results across diverse studies. Moreover, given the recent updates in WHO guidelines, there is an increased necessity to review and potentially adjust these values accordingly.

Our study further highlights the pressing need to update normality ranges for evaluating chromosomal aneuploidies in sperm, probably due to the significant decline in semen quality over time. While FISH effectively detects these abnormalities, relying on outdated internal reference set by individual laboratories risks overlooking such anomalies, potentially affecting patient outcomes. Regular updates to these standards are essential to reflect current semen quality trends and maintain precision in clinical diagnostics.

Hence, our findings, while preliminary, offer a significant contribution to comprehending the persistence of chromosomal abnormalities in male gametes. They strongly advocate for evaluating chromosomal anomalies, especially in cases of reduced sperm count.

## Supplementary information

Below is the link to the electronic supplementary material.

Supplementary file1 (DOCX 23 KB)
