# Supplementary material for: Assessment of sperm chromosomal abnormalities using fluorescence in situ hybridization (FISH): implications for reproductive potential
Source: J Assist Reprod Genet. 2024 Aug 20;41(10):2787–93. doi: 10.1007/s10815-024-03224-4 (PMC11534918; doi:10.1007/s10815-024-03224-4)
Supplement: Supplementary file 1 — Supplementary file1 (DOCX 23 KB) [file 10815_2024_3224_MOESM1_ESM.docx]

**Supplementary Material**

**Supprlementary table 1:** Probes used for FISH analysis

| **Probe** | **Lot. Number** |
| --- | --- |
| Vysis CEP X (DXZ1) Spectrum Green | 390603 |
| Vysis CEP Y (DYZ3) Spectrum Orange | 393519 |
| Vysis CEP 18 (D18Z1) Spectrum Aqua | 382934 |

**Supplemantary table 2.** Incidence of aneuploidies, diploidies and total of chromosomal anomalies in normozoospermic and samples classified in oligozoospermic, astheno/terato/asthenoteratozoospermic (ATA) and oligoasthenoteratozoospermic (OAT).

| **Bonferroni's multiple comparisons test** | **Mean frequencies** | | **p value** |
| --- | --- | --- | --- |
| **Nullisomies** |  |  |  |
| Normozoospermic vs. Oligozoospermic | 0,176 | 0,282 | 0,05 |
| Normozoospermic vs. AT | 0,176 | 0,200 | >0,9999 |
| Normozoospermic vs. OAT | 0,176 | 0,300 | 0,178 |
| Oligozoospermic vs. AT | 0,282 | 0,200 | 0,329 |
| Oligozoospermic vs. OAT | 0,282 | 0,300 | >0,9999 |
| AT vs. OAT | 0,200 | 0,300 | 0,536 |
| **Disomies** |  |  |  |
| Normozoospermic vs. Oligozoospermic | 0,319 | 0,390 | 0,463 |
| Normozoospermic vs. AT | 0,319 | 0,354 | >0,9999 |
| Normozoospermic vs. OAT | 0,319 | 0,413 | 0,586 |
| Oligozoospermic vs. AT | 0,390 | 0,354 | >0,999 |
| Oligozoospermic vs. OAT | 0,390 | 0,413 | >0,999 |
| AT vs. OAT | 0,354 | 0,413 | >0,999 |
| **Aneuploidies** |  |  |  |
| Normozoospermic vs. Oligozoospermic | 0,460 | 0,662 | *<0,0001***** |
| Normozoospermic vs. AT | 0,460 | 0,554 | 0,217 |
| Normozoospermic vs. OAT | 0,460 | 0,713 | *<0,0001***** |
| Oligozoospermic vs. AT | 0,662 | 0,554 | 0,069 |
| Oligozoospermic vs. OAT | 0,662 | 0,713 | >0,999 |
| AT vs. OAT | 0,554 | 0,713 | *0,041** |
| **Diploidies** |  |  |  |
| Normozoospermic vs. Oligozoospermic | 0,197 | 0,274 | 0,331 |
| Normozoospermic vs. AT | 0,197 | 0,248 | >0,999 |
| Normozoospermic vs. OAT | 0,197 | 0,326 | 0,137 |
| Oligozoospermic vs. AT | 0,274 | 0,248 | >0,999 |
| Oligozoospermic vs. OAT | 0,274 | 0,326 | >0,999 |
| AT vs. OAT | 0,248 | 0,326 | >0,999 |
| **Total chromosomal anomalies** |  |  |  |
| Normozoospermic vs. Oligozoospermic | 0,657 | 0,936 | *<0,0001***** |
| Normozoospermic vs. AT | 0,657 | 0,803 | *0,007*** |
| Normozoospermic vs. OAT | 0,657 | 1,040 | *<0,0001***** |
| Oligozoospermic vs. AT | 0,936 | 0,803 | *0,011** |
| Oligozoospermic vs. OAT | 0,936 | 1,040 | 0.352 |
| AT vs. OAT | 0,803 | 1,040 | *0,0004**** |

**Supplementary table 3.** Incidence of chromosome 18, X and Y in normozoospermic and samples classified in oligozoospermic, Astheno/terato/asthenoteratozoospermic (ATA) and oligoasthenoteratozoospermic (OAT).

| **Bonferroni's multiple comparisons test** | **Mean frequencies** | | **p value** |
| --- | --- | --- | --- |
| **18/18** |  |  |  |
| Normozoospermic vs. Oligozoospermic | 0,086 | 0,114 | *0,016** |
| Normozoospermic vs. AT | 0,086 | 0,119 | *0,0008**** |
| Normozoospermic vs. OAT | 0,086 | 0,126 | *0,029** |
| Oligozoospermic vs. AT | 0,114 | 0,119 | >0,999 |
| Oligozoospermic vs. OAT | 0,114 | 0,126 | >0,999 |
| AT vs. OAT | 0,119 | 0,126 | >0,999 |
| **X/X** |  |  |  |
| Normozoospermic vs. Oligozoospermic | 0,089 | 0,108 | 0.229 |
| Normozoospermic vs. AT | 0,089 | 0,080 | >0,999 |
| Normozoospermic vs. OAT | 0,089 | 0,093 | >0,999 |
| Oligozoospermic vs. AT | 0,108 | 0,080 | *0,017** |
| Oligozoospermic vs. OAT | 0,108 | 0,093 | >0,999 |
| AT vs. OAT | 0,080 | 0,093 | >0,999 |
| **Y/Y** |  |  |  |
| Normozoospermic vs. Oligozoospermic | 0,063 | 0,088 | *0,035** |
| Normozoospermic vs. AT | 0,063 | 0,066 | >0,999 |
| Normozoospermic vs. OAT | 0,063 | 0,086 | 0,608 |
| Oligozoospermic vs. AT | 0,088 | 0,066 | 0,121 |
| Oligozoospermic vs. OAT | 0,088 | 0,086 | >0,999 |
| AT vs. OAT | 0,066 | 0,086 | 0,977 |
| **X/Y** |  |  |  |
| Normozoospermic vs. Oligozoospermic | 0,053 | 0,078 | 0,049*** |
| Normozoospermic vs. AT | 0,053 | 0,087 | *0,0005**** |
| Normozoospermic vs. OAT | 0,053 | 0,106 | *0,001*** |
| Oligozoospermic vs. AT | 0,078 | 0,087 | >0,999 |
| Oligozoospermic vs. OAT | 0,078 | 0,106 | 0,332 |
| AT vs. OAT | 0,087 | 0,106 | >0,999 |
